# The Effect of Depression on Health-Related Quality of Life Is Mediated by Fatigue in Persons with Multiple Sclerosis

**DOI:** 10.3390/brainsci11060751

**Published:** 2021-06-05

**Authors:** Stephanie Rodgers, Zina-Mary Manjaly, Pasquale Calabrese, Nina Steinemann, Marco Kaufmann, Anke Salmen, Andrew Chan, Jürg Kesselring, Christian P. Kamm, Jens Kuhle, Chiara Zecca, Claudio Gobbi, Viktor von Wyl, Vladeta Ajdacic-Gross

**Affiliations:** 1 Epidemiology, Biostatistics and Prevention Institute, University of Zurich (UZH), 8001 Zurich, Switzerland; nina.steinemann@uzh.ch (N.S.); marco.kaufmann@uzh.ch (M.K.); viktor.vonwyl@uzh.ch (V.v.W.); Vladeta.Ajdacic-Gross@uzh.ch (V.A.-G.); 2 Department of Neurology, Schulthess Clinic, 8008 Zürich, Switzerland; Zina-Mary.Manjaly@kws.ch; 3 Department of Health Sciences and Technology, Eidgenössische Technische Hochschule (ETH) Zurich, 8092 Zürich, Switzerland; 4 Division of Molecular and Cognitive Neuroscience, University of Basel, 4055 Basel, Switzerland; pasquale.calabrese@unibas.ch; 5 Department of Neurology, Inselspital, Bern University Hospital and University of Bern, 3010 Bern, Switzerland; anke.salmen@insel.ch (A.S.); andrew.chan@insel.ch (A.C.); christian.kamm@luks.ch (C.P.K.); 6 Department of Neurology and Neurorehabilitation, Rehabilitation Centre Kliniken Valens, 7317 Valens, Switzerland; Juerg.kesselring@kliniken-valens.ch; 7 Neurology and Neurorehabilitation Center, Luzerner Kantonsspital, 6000 Lucerne, Switzerland; 8 Neurologic Clinic and Policlinic, Departments of Medicine, Biomedicine and Clinical Research, University Hospital and University of Basel, 4031 Basel, Switzerland; jens.kuhle@usb.ch; 9 Department of Neurology, Multiple Sclerosis Center (MSC), Neurocenter of Southern Switzerland, 6900 Lugano, Switzerland; Chiara.Zecca@eoc.ch (C.Z.); claudio.gobbi@eoc.ch (C.G.); 10 Faculty of Biomedical Sciences, Università della Svizzera Italiana (USI), 6900 Lugano, Switzerland; 11 Department of Psychiatry, Psychotherapy and Psychosomatics, Psychiatric University Hospital Zurich (PUK), 8032 Zurich, Switzerland

**Keywords:** multiple sclerosis, quality of life, depression, fatigue, longitudinal

## Abstract

The interrelations between fatigue, depression and health-related quality of life (HRQoL) in persons with multiple sclerosis (PwMS) are complex, and the directionality of the effects is unclear. To address this gap, the current study used a longitudinal design to assess direct and indirect effects of fatigue and depression on HRQoL in a one-year follow-up survey. A sample of 210 PwMS from the nationwide Swiss MS Registry was used. HRQoL was assessed using the European Quality of Life 5-Dimension 5-Level questionnaire. Path analysis on HRQoL, with fatigue and depression as predictors, was applied. Fatigue was measured by the Modified Fatigue Impact Scale (MFIS), including physical, cognitive and psychosocial subscales, and non-somatic depressive symptomatology was examined with the Beck Depression Inventory-Fast Screen (BDI-FS). Fatigue acted as a fully mediating variable (B = −0.718, SE = 0.253) between non-somatic depressive symptomatology and HRQoL. This indirect effect became apparent in the physical (B = −0.624, SE = 0.250), psychosocial (B = −0.538, SE = 0.256) and cognitive subscales (B = −0.485, SE = 0.192) of fatigue. In contrast, non-somatic depressive symptomatology did not act as a mediator. Our findings provide novel and clinically relevant longitudinal evidence showing that the debilitating effect of non-somatic aspects of depression on HRQoL was fully mediated and therefore explainable via fatigue.

## 1. Introduction

Health-related quality of life (HRQoL) is considerably reduced in persons with multiple sclerosis (PwMS) compared with both the general population and populations with other chronic diseases [1,2,3]. Previous research identified various clinical (e.g., fatigue, disability status, disease duration, progressive MS course and relapses in the last 3 months), sociodemographic (e.g., employment, sex, age, education and socioeconomic status) and psychological risk factors (e.g., depression, anxiety and perceived stress) that contribute to the impaired HRQoL in PwMS [2,3,4,5,6]. In this context, the negative impact on PwMS’s HRQoL by the two common and troublesome conditions of fatigue (affecting up to 93% at some point during the course of the disease [7,8,9,10]) and depression (lifetime prevalence 20–50% [11,12,13,14,15]) has been demonstrated in various cross-sectional studies [16,17,18,19,20,21,22] and also in a few longitudinal studies [23,24]. However, fatigue and depression show a large amount of overlapping features, challenging the investigation of their effects on HRQoL if considered independently. Few examples in the literature have addressed this topic, and the relationship generally remains poorly understood [25]. Therefore, the interrelation of fatigue and depression in HRQoL requires more consideration in PwMS.

Path analysis is a statistical method that can account for such interrelated variables by differentiating their direct and indirect effects. So far, two studies applied path analysis to examine the relation between fatigue and depression on HRQoL [2,26]. The first study, based on a Spanish sample, showed that depression mediated the relationship between some HRQoL domains, such as mental health, and fatigue in PwMS [26]. It was limited by unexpectedly low levels of depression in almost all PwMS [26]. The second study, based on a large Canadian sample, demonstrated that the relation of depression on HRQoL was almost equally split into a direct and indirect association through its influence on fatigue or anxiety. This finding indicated that the influence of depression on HRQoL may be partly mediated by its effects on fatigue [2]. Because these two studies were cross-sectionally designed, causal conclusions were, however, limited. Longitudinal data would provide the possibility of examining the direction of the relations, but despite the importance of the issue, no study with PwMS has applied a longitudinal analysis on the interrelation between fatigue and depression on HRQoL up to now.

Moreover, studies subdividing fatigue into its different cognitive and physical components showed that the relation between depression and fatigue in PwMS was more pronounced for cognitive fatigue in comparison with physical fatigue [27,28]. This finding suggests that specific components of fatigue should be considered, as it is a heterogeneous construct with somatic and non-somatic symptoms [8]. Additionally, depression is an inherently heterogeneous construct comprising non-somatic as well as somatic components. Consequently, specific instruments assessing depression under the exclusion of overlapping somatic symptoms were thus developed to counteract a potentially biased overestimation of depression in PwMS [29,30,31,32] (somatic symptoms: loss of energy, sleep problems, irritability, appetite problems, fatigue and loss of interest in sex; cognitive or affective symptoms: sadness, pessimism, past failure, loss of pleasure, guilty feelings, punishment feelings, self-dislike, self-criticism, suicidal ideation, crying, agitation, loss of interest, indecisiveness, worthlessness and concentration problems [33]). Therefore, it is important that longitudinal analyses on fatigue and depression in PwMS take into account that both entities have somatic and non-somatic phenomena.

Our objective was to examine the directionality of fatigue and depression on HRQoL in a national database of PwMS by applying a longitudinal path analysis. First, we hypothesized that the influence of depression on HRQoL would be mediated via fatigue. In doing so, we focused on the non-somatic aspects of depressive symptomatology in order to distinguish them from MS-related fatigue. Second, we hypothesized that the impact via the cognitive fatigue subscale on HRQoL will be more pronounced compared with the physical and psychosocial fatigue subscales. 

## 2. Materials and Methods

### 2.1. Study Sample and Data Collection

The Swiss MS Registry (SMSR) is a patient-centered prospective study assessing the life circumstances of adult PwMS and their relatives and proxies in Switzerland (http://www.Clinical-Trials.gov, accessed on 4 June 2021, identifier: NCT02980640). This observational study was initiated and is funded by the Swiss MS Society, and it is based on self-reported data. Study recruitment started in June 2016 and is still ongoing (*n* = 2465, status quo 19 April 2021). Participation is possible via an online system or via paper-pencil versions, and all surveys are offered in German, French or Italian [34,35]. The SMSR was approved by the ethics committee of the Canton of Zurich (PB-2016-00894; BASEC-NR 2019-01027), and the participants signed written informed consent after being informed about the study’s procedure and aims in writing [34].

**Figure 1:**
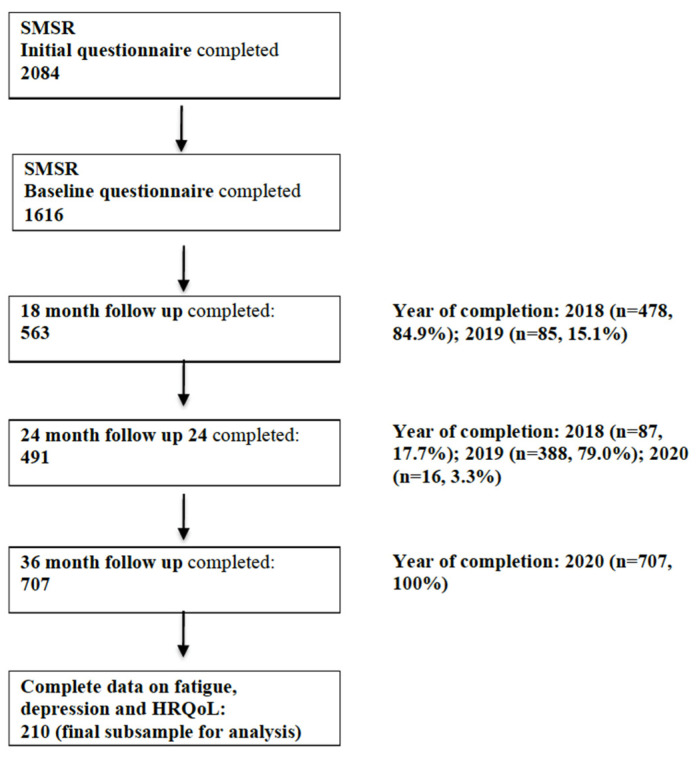
Flow chart describing the study sample of the Swiss Multiple Sclerosis Registry (numbers reflect numbers of persons; status quo: 8 June 2020). Abbreviations: SMSR = Swiss MS Registry and HRQoL = Health-related quality of life.

In total, 563, 491 and 707 participants filled out the 18-month, 24-month and 36-month follow-up surveys, respectively (status quo as per 8 June 2020; Figure 1). For the current study, we focused only on PwMS with complete data in all three follow-up surveys assessing depression, fatigue and HRQoL, leading to a final subsample of *n* = 210. The reduction of the sample was mainly due to the restricted availability of the depression and fatigue instruments. 

### 2.2. Measurements

Information on the basic sociodemographic and health-related variables was obtained from the preliminary SMSR assessments (labelled as “initial” and “baseline”) and comprised the following factors: sex, age, education level, occupational status, civil status and smoking status.

Information on HRQoL, the primary outcome, was collected twice: in the 18-month (t0) and 36-month follow-up surveys (t1). HRQoL was assessed using the European Quality of Life 5-Dimension 5-Level questionnaire (EQ-5D-5L). This questionnaire covered the following five dimensions: (1) mobility, (2) self-care, (3) usual activities, (4) pain or discomfort and (5) anxiety or depression [36,37]. It provided a visual analogue scale (EQ-VAS) and an estimation of a single utility figure (also referred to as the EQ-5D-index). For the current study, the French value set was used [38,39]. The EQ-VAS ranged from 0 (worst imaginable health) to 100 (best imaginable health), and the EQ-5D-index was rescaled from 0 (worst health) to 100 (best health) [40].

The following information was taken from the 24-month follow-up survey.

The Modified Fatigue Impact Scale (MFIS) is a modified form of the Fatigue Impact Scale [41] and assesses fatigue in terms of physical, cognitive and psychosocial functioning apart from an overall fatigue sum score. This questionnaire has been recommended for research purposes and in clinical practice [42]. It consists of 21 items, and individual subscales can be generated by calculating the sum of a specific set of items. The MFIS has a Cronbach’s alpha of 0.92 [43]. Clinically relevant fatigue was based on the MFIS sum score cut-off greater than or equal to 38 as recommended elsewhere [43].

The seven-item Beck Depression Inventory-Fast Screen (BDI-FS) [44] assessing current subjective burden resulting from depression was applied. This self-rating questionnaire has been specifically validated for use with PwMS [31]. The BDI-FS captures depression (time span: past two weeks) under the exclusion of somatic features. Thus, it counteracts the potentially biased overestimation of depression prevalence in PwMS due to confounding with MS-related symptoms, such as fatigue. Clinically relevant non-somatic depressive symptomatology was defined as a BDI-FS sum score greater than or equal to 4, as this cut-off showed sensitivity between 0.97 and 1.00 and specificity between 0.79 and 0.99 [45,46].

The Expanded Disease Status Scale (EDSS) ranges from 0 to 10, with low values indicating no physical disability or disturbance and high values indicating greater disability. Information on the EDSS was derived from the self-reported disability status scale (SRDSS), a proxy measure based on the same cohort [47]. EDSS proxy values were dichotomized into high (defined as scores ≥4) versus low (defined as scores <4) disability.

In addition, the MS disease phenotypes and the time since MS diagnosis were collected in the two-year follow-up SMSR survey. MS disease phenotypes were dichotomized into two groups: (1) relapsing-remitting MS (RRMS) versus (2) progressive MS courses (secondary progressive MS (SPMS), primary progressive MS (PPMS) and transitions between MS courses).

### 2.3. Statistical Analysis

Descriptive statistics were provided by absolute and relative frequencies or medians (interquartile range (IQ) 25th percentile (PI); 75 PI). Univariable and multivariable associations between the depression sum scores and the fatigue sum scores as potential mediator (M) or suppressor (S) variables on the metric HRQoL were computed using regression analysis (unstandardized coefficient B, standardized coefficient β and odds ratios (ORs) with 95% confidence intervals (CIs)). The following criteria of Baron and Kenny were applied in order to check the variables in terms of potential Ms and if partial or full mediation occurred: (1) independent variable (IV) on dependent variable (DV) significant; (2) IV on M/S significant; (3) M/S on DV significant; and (4) a multiple regression of IV and M/S on DV, in which M/S remained significant and IV was either no longer significant (full mediation) or still significant but reduced in its strength (partial mediation) [48].

Statistical significance was defined as a *p*-value < 0.05. The selection of relevant independent variables and covariates was based on a combination of theoretical evidence and a backward selection of variables (*p*-values: in = 0.05; out = 0.10). Goodness-of-fit according to Cohen [49] was used for judging the overall model.

Separate path models with HRQoL as the DV were computed, treating (1) non-somatic depressive symptomatology as the IV and fatigue as M and (2) fatigue as the IV and non-somatic depressive symptomatology as M. An additional path model was computed, differentiating between the fatigue subscales. Considering that the EQ-5D-index is composed of physical components, which are similarly captured by the MFIS, we used the EQ-VAS, assessing subjective burden as the principal outcome. Unstandardized regression coefficients (B) were computed as recommended elsewhere [50]. Indirect effects, implying that an IV caused the M and the M caused the DV, were considered significant if the 95% CI did not contain the value 1.0 [51], based on Sobel’s method [52].

The path models were conducted using Mplus for Macintosh, version 8 [53]. A descriptive analysis and regression analysis were performed in IBM SPSS Statistics for Macintosh, version 25.0 [54] and Stata software for Macintosh, version 13.1 [55].

## 3. Results

### 3.1. Descriptive Analysis

The sociodemographic characteristics of the sample (*n* = 210) of PwMS living in Switzerland are presented in Table 1. Participants had a mean age of 51.0 years and were predominantly female (71%), highly educated (62%), working (68%) and married or in a registered partnership (58%). In total, 70% of participants reported having an RRMS, while the other larger groups were composed of secondary SPMS (17%) and PPMS courses (8%). The mean MS disease duration was 11.0 years. A proportion of 70% had EDSS proxy values [47] of 3.5 maximum, while around 30% had higher scores, indicating higher levels of disability. Around a third of the sample reached the critical score level that was considered to reflect clinically relevant fatigue symptomatology, derived from the MFIS scores. The HRQoL scores showed medians of 80 (EQ-VAS) and 71 (EQ-5D-index) for both time points. Considering the BDI-FS scores, approximately a quarter of the sample fulfilled the criteria for a clinically significant depressive symptomatology. Missing values concerning potential covariates were between 0.5% and not more than 3.3% per variable (Table 1).

### 3.2. Regression and Path Analysis

Table 2 provides the univariable and multivariable associations of the first mediation model, using depression as the IV and fatigue as M, which met the criteria according to Baron and Kenny. On the one hand, high BDI-FS depression scores (IV) were significantly associated with higher MFIS fatigue sum scores (M) and, on the other hand, reduced EQ-VAS HRQoL scores (DV). Reduced EQ-VAS scores were also significantly associated with high MFIS fatigue sum scores (M). However, as soon as both the IV and M were inserted into the model, the effect of the IV on the DV failed to reach significance, while the effect of M on the DV remained significant. These results indicated a full mediation of the IV on the DV via the M variable, according to the Baron and Kenny criteria (Table 2). Based on the backward selection of variables, all analyses were adjusted for EQ-VAS at t0, sex, age, civil status, EDSS proxy values and MS disease courses. The R2 for the overall model was 0.53 (adjusted = 0.51), indicating high goodness-of-fit according to Cohen [49]. On the contrary, the Baron and Kenny criteria were not fulfilled in the second mediation model, where the MFIS fatigue sum score was set as the IV variable and the BDI-FS depression scores were the M variable, thus suggesting that the association between fatigue and HRQoL was not explainable via depression (data not shown).

**Figure 2:**
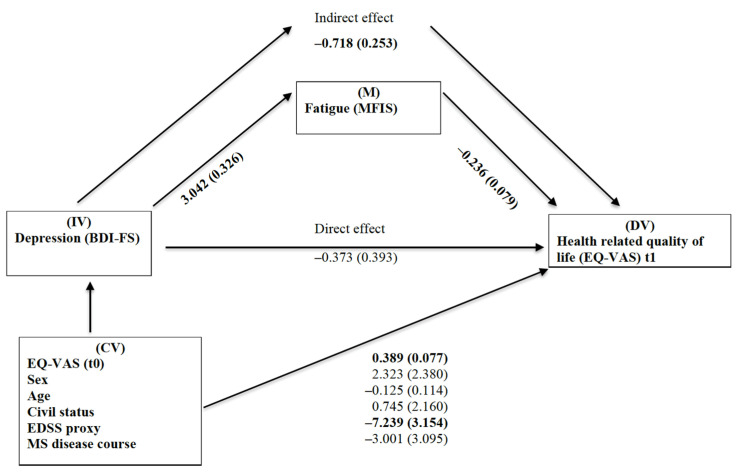
Path analysis of the psychological predictors of depression and fatigue for health-related quality of life (EQ-VAS) in persons with MS (*n* = 206). The values on the arrows are unstandardized parameter estimates with standard errors. Bold indicates significance (*p* < 0.05). Abbreviations: IV = independent variable; DV = dependent variable; M = mediator; CV = covariates; MS = multiple sclerosis; BDI-FS = Beck Depression Inventory-Fast Screen; MFIS = Modified Fatigue Impact Scale; HRQoL = health-related quality of life; EQ-VAS = European Quality of Life 5-Dimension 5-Level version visual analogue scale; and EDSS = Expanded Disease Status Scale.

Figure 2 presents the unstandardized parameter estimates of the first path model, including the overall MFIS fatigue sum score. The estimates indicated a full mediation of the BDI-FS depression scores on EQ-VAS HRQoL scores via the MFIS fatigue sum scores, showing that the effect of non-somatic depressive symptomatology on HRQoL was split into a non-significant direct path (B = −0.373, SE = 0.393) and a significant indirect path (B = −0.718, SE = 0.253) via fatigue.

The indirect effects from three separate path models differentiated by the MFIS fatigue subscales of physical, cognitive and psychosocial functioning are presented in Table 3. The physical subscale (B = −0.624, SE = 0.250) and the psychosocial subscale (B = −0.538, SE = 0.256) showed the strongest indirect effects between the BDI-FS depression scores and a reduced EQ-VAS HRQoL, whereas the effect via the cognitive subscale was slightly weaker (B = −0.485, SE = 0.192). However, all three indirect effects were significant.

## 4. Discussion

Our longitudinal results suggest that fatigue fully mediates the association between non-somatic depressive symptomatology and HRQoL in PwMS. This indirect mediation effect on HRQoL became apparent in all three fatigue subscales, with the most severe impact occurring in the physical and psychosocial fatigue subscales followed by the cognitive fatigue subscale. Therefore, the debilitating influence of the non-somatic aspect of depression on HRQoL was explained by indirect effects exerted by fatigue.

The finding of the indirect effect between depression and HRQoL through fatigue supports our first hypothesis, predicting that the influence of depression on HRQoL would be mediated by fatigue. So far, two cross-sectional studies previously considered the interrelation between fatigue and depression on HRQoL by path analysis. Our findings were in accordance with a Canadian study based on a large sample of PwMS reporting the same relation [2]. Moreover, we were able to substantiate the evidence by adopting a longitudinal approach, which is more suitable than cross-sectional analyses when hypothesizing causal relationships between IVs and DVs and applying path analysis models. Notably, by capturing the non-somatic aspects of depression (i.e., referring to the cognitive and affective dimensions), we avoided a potential overestimation bias, which inevitably appears when including somatic depression features in PwMS [32]. Hence, this different way of assessing depression may also explain why we detected a full mediation, in contrast to the aforementioned Canadian study showing partial mediation [2]. Finally, the differentiation by the three fatigue subscales was also a new clinically relevant aspect of the current study.

In contrast, the path analysis study based on a Spanish sample had an inversed theoretical framework by hypothesizing that the effect of an HRQoL domain (mental health) on fatigue was mediated by depression in PwMS [26]. This was explained by the fact that PwMS with fatigue experience decreased cognitive function, promoting depressive symptoms which are followed again by increased self-perceived fatigue, pointing to a vicious cycle [26,56]. The etiology of fatigue in MS is not precisely understood and is thought to be heterogeneous, with different underlying pathophysiological mechanisms leading to the same downstream symptomatology [8]. While some authors see fatigue as a direct consequence of the MS disease process itself (e.g., [57]), others interpret it as a corollary effect of depression or sleep disturbances (e.g., [30]). A study directly comparing four models predicting fatigue in PwMS highlighted depression and sleep disturbances as strong predictors of fatigue, apart from disease severity [58]. Therefore, our hypothesis that the disabling nature of non-somatic depressive symptomatology may contribute to fatigue in PwMS seems highly plausible, even though it is possible that it is restricted mainly to the secondary aspect of fatigue [59].

In addition, studies not applying longitudinal path analysis reported worse HRQoL for PwMS with depression or fatigue (e.g., [3,17,21,23,24]). In line with these studies, we previously showed in a cross-sectional analysis that depression belonged to the three leading symptoms for HRQoL losses in persons with RRMS courses [16]. In addition, our present study confirmed the detrimental effect of depression on HRQoL for the non-somatic depressive symptomatology.

The strength of the relationship between depression and fatigue was more pronounced for cognitive fatigue in comparison with physical fatigue in PwMS in previous research [27,28]. A cytokine-mediated pathogenesis of depression and cognitive fatigue was suggested as a possible explanation for this relation [27]. However, we were not able to identify a differential relationship of any fatigue subscale regarding non-somatic depressive symptomatology and the HRQoL outcome. In fact, all three fatigue subscales were statistically associated with HRQoL, even though the most severe impact occurred in the physical and psychosocial fatigue subscales, followed by the cognitive subscale. Thus, our second hypothesis, predicting the strongest impact via the cognitive fatigue subscale on HRQoL, was not supported. This finding may reflect the intricate multifactorial relationship between the physical, as well as cognitive, and psychosocial components, whose interaction transposes then into an inextricable main effect. In fact, up regulated inflammatory activity, which may lead to depressive symptomatology by altering neuroinflammatory as well as monoamine and glutaminergic metabolism pathways in the brain, occurred in response to psychosocial as well as cognitive and physical stressors or pathogens [60].

Our study contributes to a better directional understanding of the overlapping and heterogeneous constructs of fatigue and depression on HRQoL in PwMS by considering their specific components and by disentangling their direct and indirect effects in a longitudinal design. From a clinical point of view, the overlap of depression and fatigue in PwMS depicts a particular therapeutic challenge [61]. Hence, it is of clinical importance to differentiate between the somatic components of depression that may appear to be preponderant due to their MS-related physical symptomatology and some subtle non-somatic signs indicating mental affliction. By doing so, disadvantageous pharmacological interventions based on the overinterpretation of somatic signs (e.g., “drug-induced low-mood”) can be avoided. Hence, for these overlapping cases, Brenner and Piehl [61] suggested that it is prudent to first treat depressive symptoms before fatigue due to the better evidence for pharmacological interventions in depression. An alternative therapeutic avenue for reducing fatigue in PwMS that is gaining increasing attention is the use of contemplative techniques, such as mindfulness-based interventions. These have shown potential for treating fatigue in MS [62,63,64] and have the additional benefit that specific programs exist that focus on depression (e.g., mindfulness-based cognitive therapy (MBCT) [65]).

### Limitations

Our study has some limitations that warrant discussion. We adopted a statistical approach based on particular mediation criteria concerning the relations of depression, fatigue and HRQoL, even though these criteria earned some criticism [66] and additional interactions and causal relations between these variables may occur. Additionally, both variants (1) that fatigue may include depression, but (2) it can also occur independently [58] are certainly plausible. Thus, our approach does not mirror the entire heterogeneity; rather, it reflects one possible occurrence of the overlapping constructs of depression and fatigue, partly referring to secondary fatigue. When interpreting the results, it is also important to consider that fatigue is a broad construct with some facets that are also present in non-somatic depressive symptomatology. Therefore, one might have anticipated that the non-somatic form of depression may be more probable to be represented in fatigue than vice versa in the tested path model relations. Nevertheless, non-somatic depressive symptomatology was not only reflected in the cognitive fatigue component but also in the physical and psychosocial one, which did not confirm this assumption. Moreover, we cannot exclude the possibility that the physical fatigue subscale at least in part contains the commonly captured somatic aspects of depression. A similar difficulty pertains to the unavoidable overlap of both the concepts of depression and HRQoL. This overlap may result in poorer judgment of the HRQoL, depending on the level of depression severity as was discussed by other authors [30]. Assessing the HRQoL following the remission of depression or rated by other persons are possibilities for avoiding this kind of negative bias between depression and HRQoL [30]. However, we judged this bias as marginal for the current study, as different time frames between depression screening and HRQoL assessment were applied. In addition, the EQ-VAS captures the current daily condition, which can vary. Considering the highly stable EQ-VAS medians between the two separate surveys, however, this is not a serious limitation. Another limitation of the study is that the t0-HRQoL was assessed six months before the BDI-FS and MFIS. Therefore, we cannot exclude that further factors occurring in the time span might have contributed to some t1-HRQoL shifts. Moreover, antidepressant and fatigue treatments were not included in the final path analysis. The interrelations of this variable with depression, fatigue and HRQoL are ambiguous, complex and would require separate path analyses. However, the effects found in the current study remained stable under the inclusion of antidepressant treatment as an additional covariate in an exploratory manner. Then, sleep disturbances and cognitive dysfunction were not considered in the current analysis, despite their relationship with both fatigue and depression [58]. We only focused on the two particularly troublesome factors of fatigue and depression, although many other causal pathways predicting HRQoL in PwMS are possible. Additionally, generalizability might be limited, as the included subsample with complete data on depression, fatigue and HRQoL led to an unavoidable selection bias with older, highly educated, often-employed participants with rather good physical functioning. Finally, the novel clinically relevant entity referring to “long COVID syndrome” was not yet ongoing in the data collection used for the current analysis, but it needs special attention in future studies.

## 5. Conclusions

The current study provides novel longitudinal evidence by demonstrating that the impact of non-somatic depressive symptomatology on HRQoL was explainable by an indirect effect via fatigue. Here, all fatigue subscales (physical, psychosocial and cognitive fatigue) mediated this relation. Although this study focused on how the effect of depression on HRQoL is mediated by fatigue, it is important to keep in mind that fatigue in PwMS can occur independently of depression. For PwMS with both depression and fatigue, it is of clinical importance that pharmacological and non-pharmacological interventions are appropriately considered regarding both conditions. For example, pharmacological treatment of underlying depression and mindfulness-based interventions may have a positive effect not only on depression but also on fatigue. In particular, regarding physical and psychosocial fatigue, which is reflected in difficulty participating in social or outdoor activities, non-pharmacological interventions such as the enhancement of physical activity might be feasible and potentially promising. A better understanding of the underlying mechanisms of the interrelations between depression, fatigue and HRQoL is needed and should be targeted in future research.

## Figures and Tables

**Table 1:** Demographic and disease-related characteristics of the Swiss MS Registry sample (*n* = 210).

Sample Characteristic	*n* = 210
Sociodemographics	
Sex	
Women	150 (71.4%)
Men	60 (28.6%)
Age (median (IQR))	50.5 (44.0; 58.0)
Education ^1, a^	
Low	78 (38.2%)
High	126 (61.8%)
Occupational status ^b^	
Working	141 (67.5%)
Not working	68 (32.5%)
Urbanicity ^2^	
Urban	192 (91.4%)
Urban to rural	3 (1.4%)
Rural	15 (7.1%)
Civil status	
Married or registered partnership	121 (57.6%)
Other	89 (42.4%)
Clinical MS-related MS Disease Course ^c^	
CIS	2 (1.0%)
RRMS	146 (69.9%)
PPMS	17 (8.1%)
SPMS	36 (17.2%)
Transition or other type	8 (3.8%)
Dichotomized MS Disease Course (Excluding CIS) ^c^	
RRMS	146 (70.5%)
Progressive MS (PPMS, SPMS, Transition)	61 (29.5%)
Time since MS Diagnosis ^d^ (median (IQR))	11.0 (6.0; 17.0)
Disease Modifying Treatment ^e^ (current, past 6 months)	
Yes	146 (71.2%)
No	59 (28.8%)
Bouts ^f^ (current, past 6 months)	
Yes	15 (7.8%)
No	177 (92.2%)
Proxy Measure to Estimate EDSS ^g^	
EDSS 0–3.5	147 (70.3%)
EDSS 4–6.5	45 (21.5%)
EDSS ≥ 7	17 (8.1%)
Fatigue Sum Scores (MFIS)	
Clinically relevant fatigue	71 (33.8%)
No clinically relevant fatigue	139 (66.2%)
Overall sum score (median (IQR))	30.0 (13.8; 42.0)
Cognitive subscale (median (IQR))	11.0 (5.0; 18.0)
Physical subscale (median (IQR))	15.0 (5.8; 20.0)
Psychosocial subscale (median (IQR))	3.0 (1.0; 4.3)
Health-Related Quality of Life	
EQ-VAS (18-month follow-up survey) (median (IQR))	80.0 (65.0; 90.3)
EQ-5D-index (18-month follow-up survey) ^h^ (median (IQR))	71.1 (50.5; 92.9)
EQ-VAS (36-month follow-up survey) (median (IQR))	80.0 (60.0; 90.0)
EQ-5D-index (36-month follow-up survey) ^i^ (median (IQR))	71.4 (47.2; 91.0)
Clinical Depression-Related BDI-FS	
Clinically relevant depressive symptomatology	53 (25.2%)
No clinically relevant depressive symptomatology	157 (74.8%)
Overall sum score (median (IQR))	1.0 (0.0;4.0)
Antidepressants	
Yes	16 (7.6%)
No	197 (92.9%)
If yes (*n* = 16)	
Selective serotonin reuptake inhibitors (SSRIs)	5 (31.2%)
Serotonin–norepinephrine reuptake inhibitors (SNRIs)	5 (31.2%)
Serotonin antagonist and reuptake inhibitors (SARIs)	1 (6.3%)
Tricyclic antidepressants (TCAs)	1 (6.3%)
Herbal antidepressants (St. John’s wort)	2 (12.5%)
Detailed information missing	2 (12.5%)
Psychotherapy	
Yes	32 (15.2%)
No	178 (84.8%)
Consumption of Psychoactive Substances	
Smoking Status	
Still smoking	35 (16.7%)
No	175 (83.3%)

Results are shown as a number (percentage) or median (range or interquartile range (25PI; 75PI)). Abbreviations: MS = multiple sclerosis; IQR = interquartile range; CIS = clinically isolated syndrome; RRMS = relapsing-remitting MS; SPMS = secondary progressive MS; PPMS = primary progressive MS; EDSS = Expanded Disease Status Scale; MFIS = Modified Fatigue Impact Scale; EQ-5D = the European Quality of Life-5 Dimensions; FU = follow-up; and BDI-FS = Beck Depression Inventory-Fast Screen for medical patients. ^1^ High: High school or higher. ^2^ Based on the Federal Statistical Office of Switzerland. ^a^ *n* = 6; ^b^ *n* = 1; ^c^ *n* = 1; ^d^ *n* = 7; ^e^ *n* = 5; ^f^ *n* = 18; ^g^ *n* = 1; ^h^ *n* = 1; ^i^ *n* = 1.

**Table 2:** Univariable and multivariable results of the variables meeting the mediator criteria according to Baron and Kenny (1986) [48], adjusted ^1^ (*n* = 206) ^2^.

						Effects on EQ-5D (DV)											
IV	M	Effect of IV on M				Direct Effect of IV on DV			Effect of M on DV					Effect of IV on DV after Inclusion of M to the Model			
		Estimate				Estimate			Estimate					Estimate			
		B	SE	β	*p-*Value	B	SE	β	*p-*Value	B	SE	β	*p-*Value	B	SE	β	*p-*Value
**BDI-** **FS** **Sum Score**	**MFIS Sum Score**	**1.926**	**0.326**	**0.345**	**<0.001**	**−0.828**	**0.378**	**−0.125**	**0.030**	**−0.265**	**0.075**	**−0.224**	**<0.001**	−0.373	0.402	−0.056	0.355

BDI-FS = Beck Depression Inventory-Fast Screen for medical patients; MFIS = Modified Fatigue Impact Scale; IV = independent variable; M = mediator; DV = dependent variable; B = unstandardized coefficient; SE = standard error; β = standardized coefficient; EQ-5D = the European Quality of Life-5 Dimensions; and EDSS = Expanded Disease Status Scale. ^1^ Adjusted for EQ-VAS (t0) sex, age, civil status EDSS proxy values and MS disease course. ^2^ From the original sample of *n* = 210, 4 (1.9%) persons were excluded due to missing values on the covariates, leading to a final sample of *n* = 206. Bold indicates *p* < 0.05 significance level.

**Table 3:** Indirect effects by fatigue subscales, derived from three separate path analyses of the predictors depression and fatigue, on health-related quality of life (EQ-VAS) in persons with MS (*n* = 206) ^1^.

MFIS Subscale	B	SE
Physical subscale	**−0.624**	**0.250**
Psychosocial subscale	**−0.538**	**0.256**
Cognitive subscale	**−0.485**	**0.192**

B = unstandardized coefficient and SE = standard error. ^1^ Adjusted for EQ-VAS (t0), sex, age, civil status, MS disease course (dichotomized) and EDSS proxy values. Bold indicates *p* < 0.05 significance level. Abbreviations: MFIS = Modified Fatigue Impact Scale; EQ-VAS = European Quality of Life 5-Dimension 5-Level version visual analogue scale; and EDSS = Expanded Disease Status Scale.

## Data Availability

Data are available from the authors upon reasonable request.
